# Enantiopure Derivatives of Aza-Baylis-Hillman Adducts by Subsequent S_N_′-S_N_′ Reactions of Acylcarbamates Bearing a Chiral Auxiliary 

**DOI:** 10.3390/molecules14082824

**Published:** 2009-07-30

**Authors:** Gianluca Martelli, Eleonora Marcucci, Mario Orena, Samuele Rinaldi

**Affiliations:** Dipartimento I.S.A.C. - Università Politecnica delle Marche, Via Brecce Bianche - I-60131 Ancona, Italy; E-mail: srinaldi@univpm.it (S.R.)

**Keywords:** chiral auxiliary, aza-Baylis-Hillman, rearrangement

## Abstract

Reactions of (4*S*,5*R*)-1-(3,4-Dimethyl-2-oxo-5-phenylimidazolidine)carbonyl-isocyanate (**4**) with appropriate Baylis-Hillman adducts **5** gave the corresponding acyl carbamates **6,7** as equimolar diastereomeric mixtures. These mixtures were treated with DABCO, to afford with moderate diastereoselection easily separable [2-(3",4"-dimethyl-2"-oxo-5"-phenylimidazolidine-1-carboxamido)(aryl)methyl]acrylates **8** and **9**.

## Introduction

Biological processes are mainly governed by the interaction of peptides with macromolecular receptors. For these interactions to be successful, the three-dimensional conformation of the peptide chain is of crucial importance, and considerable efforts have been directed towards improving the pharmacological properties of natural peptides by structure modifications of the amino acid constituents [1,2,3]. Thus, in recent years research has largely focused on the incorporation of stereochemically constrained amino acids into peptides, with the aim of effectively reducing the populations of possible peptide chain conformations, enhancing in turn potency, receptor selectivity and pharmacokinetic properties [4,5,6]. In addition, modifications which decrease conformational mobility are useful in order to gain insight into the relationship between the biological activity and the three dimensional topology, new procedures aimed to enforce conformational restrictions in peptides are welcome. In this field, we sought to prepare enantiomerically pure derivatives of α-methylene β-amino acids [7,8,9,10], which can be also obtained by aza-Baylis-Hillman reactions [11,12]. In fact, these compounds deserve interest in their own right due to the conformational restriction of the double bond [13]. In addition, the functionalization of the double bond leads to an α-quaternary centre, also able to induce conformational restrictions [14].

## Results and Discussion

**Scheme 1:**
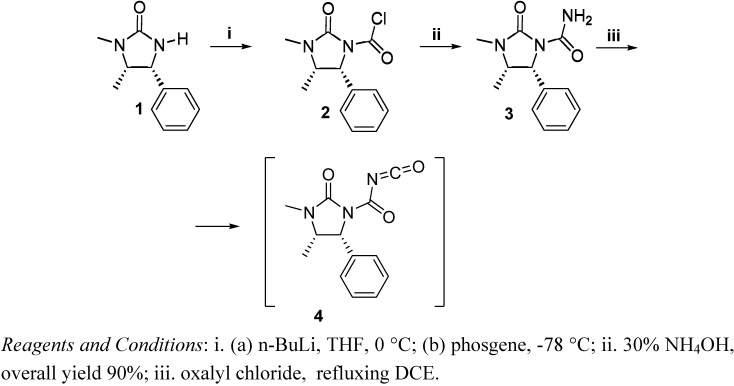
Synthesis of (4*S*,5*R*)-1-(3,4-Dimethyl-2-oxo-5-phenylimidazolidine)carbonyl-isocyanate (**4**).

The use of chiral auxiliaries for controlling the stereochemistry of numerous reactions is well established. Thus, in recent years imidazolidin-2-one **1** and its enantiomer, easily obtained starting from either enantiomer of ephedrine [15], were employed as versatile chiral auxiliaries in a lot of synthetically useful reactions [16,17,18]. In fact, on addition of a solution of the lithium anion of the compound **1** in THF to a solution of phosgene in toluene at -78 °C, the corresponding chloride **2** was obtained [18], which was subsequently converted into the derivative **3** by reaction with a 30% solution of ammonia (Scheme 1) [19]. Following the literature method, compound **3** was converted into the corresponding acyl isocyanate **4** [20].

**Scheme 2:**
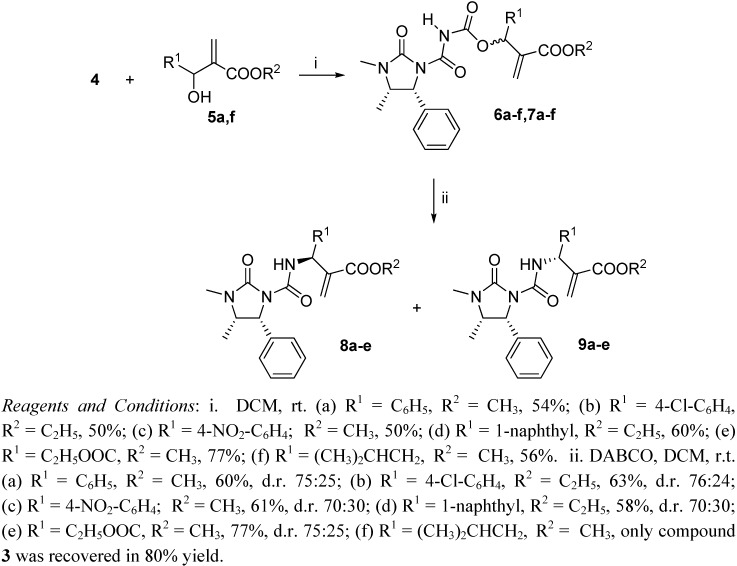
Synthesis of [2-(3",4"-dimethyl-2"-oxo-5"-phenylimidazolidine-1-carbox-amido)(aryl) methyl] acrylates **8** and **9**.

The solution containing compound **4** was immediately treated with the Baylis-Hillman adducts **5a-f**, leading in good yield to the acyl carbamates **6,7** as nearly equimolar diastereomeric mixtures. Since these compounds were hard to separate, only small samples could be isolated for analytical purposes and they were assigned as **6** or **7** on the basis of their *R*_f_, exclusively (Scheme 2). Then, the mixtures of acyl carbamates **6,7** were treated with DABCO [21], affording in moderate yield diastereomeric mixtures of compounds **8** and **9** which in turn were easily separated by silica gel chromatography. The configurations at the newly formed stereogenic centre were assigned as *S* for the major component of the reaction mixture and as *R* for the minor one, on the basis of both mechanistic considerations and ^1^H-NMR data. 

**Scheme 3:**
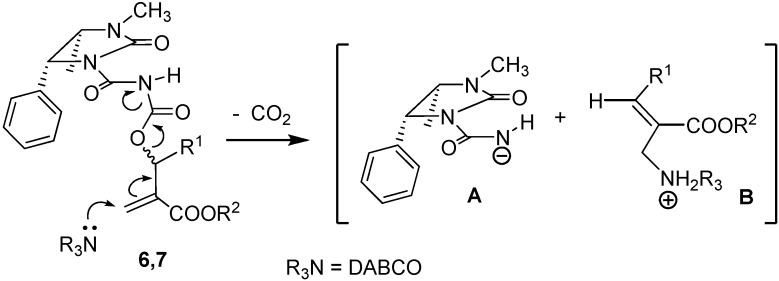
Formation of the intermediates **A** and **B** in the first S_N_' pathway.

Thus, having observed for the reaction leading to both **8** and **9** an interesting asymmetric induction, that is attractive owing to the easy separation of the diastereomeric products **8** and **9**, we first assigned the configuration of the newly formed stereogenic centre on the basis of mechanistic considerations. In fact, in the first step of the reaction of acylcarbamates **6**,**7**, DABCO attacks the conjugate double bond and the first products are the anionic species **A** and the cationic species **B**, both resulting from the initial S_N_′ pathway, involving loss of carbon dioxide, irrespectively from the configuration at C-1' of the starting acyl carbamate (Scheme 3).

**Scheme 4:**
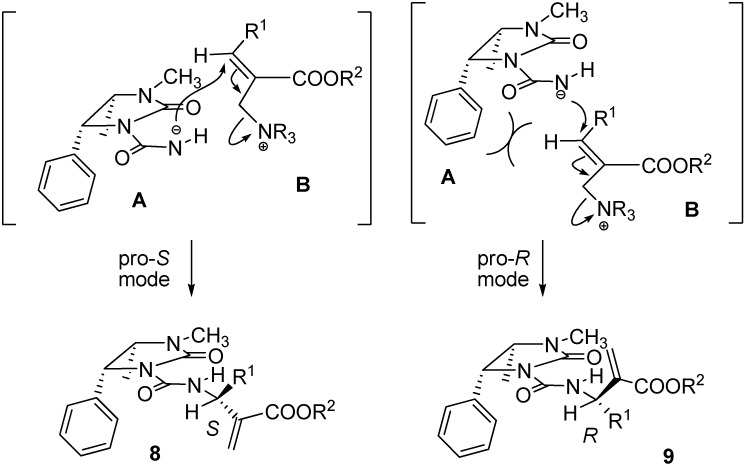
Preferential formation of compound **8** due to steric interaction in the second S_N_' pathway.

The asymmetric induction arises from the second S_N_**'** pathway, in which the anion A attacks the cationic species **B** leading to the ureas **8** and **9**. As it appears from Scheme 4, the attack proceeding *via* a pro-*S* mode is free from steric interactions, whereas in the attack occurring in the pro-*R* mode steric interaction results from the phenyl group and **B**. Thus, the two transition states leading to **8** and **9**, respectively, are different in energy and the less demanding **8** is favoured with respect to **9**.

Mechanistic considerations are supported also by the chemical shift pattern observed for the olefinic protons for both **8** and **9**. In fact, since H-1' in the preferential conformation must be co-planar with the sp^2^ urea system, the C-3 olefinic protons in **8**, displaying the *S*-configuration at carbon atom of methine group attached to C-2, experience the shielding effect of the phenyl group of the auxiliary, and are observed upfield with respect to the same protons in **9**, having the *R*-configuration, where this effect is missing. On the other hand, the chemical shift of H-1' remains unchanged in both diastereomers (Table 1). Eventually, the configurational assignment at C-1' is in agreement with the *R*_f_ values, observed for compounds **8** and **9**, these latter being more polar owing to the less compact conformation.

We can also tentatively explain the total lack of reactivity observed for the acylcarbamates **6f** and **7f**. In this case, the intermediate **A** surely forms, owing to the recover of compound **3**, but further S_N_' process cannot occur owing to the low electrophilicity of C-3, in which the alkyl group significantly decreases the electron-withdrawing effect of both the ester and the ammonium ion, thus preventing the second reaction.

**Table 1:** Significant ^1^H-NMR data for both olefinic protons and H-1' in compounds **8** and **9.**

Compound	δ(H_cis_)	δ(H_trans_)	δ(H-1')
**8a**	6.29	5.82	5.26
**9a**	6.36	5.91	5.28
**8b**	6.31	5.81	5.26
**9b**	6.37	5.89	5.27
**8c**	6.38	5.92	5.26
**9c**	6.43	5.97	5.28
**8d**	6.38	5.90	5.28
**9d**	6.46	6.00	5.32
**8e**	6.31	5.85	5.26
**9e**	6.37	5.92	5.28

In conclusion, starting from the Baylis-Hillman adducts 5, we were able to prepare chiral derivatives of the aza-Baylis-Hillman adducts by using two successive S_N_' steps. These compounds can be further elaborated by removing the chiral auxiliary, leading to monomers useful for preparing new β-foldamers, and work along this line is currently underway.

## Experimental

### General

Melting points (uncorrected) were measured on a Electrothermal IA 9000 apparatus. NMR spectra (200 MHz for ^1^H, 50 MHz for ^13^C, chemical shifts as ppm in the δ scale, coupling constants *J* in Hertz) were recorded at 25 °C in CDCl_3_ solutions on a Varian Gemini 200 spectrometer. Diastereomeric ratios were determined with a liquid chromatography Agilent Technologies HP1100 equipped with a Zorbax Eclipse XDB-C8 Agilent Technologies column (flow rate 0.5 mL/min) and equipped with a diode-array UV detector (220 and 254 nm). Enantiomeric purity of all compounds was determined ≥98% by using a Chiralcel OD column. Acetonitrile and methanol for HPLC were purchased from a commercial supplier. Samples were prepared by diluting 1 mg in 5 mL of a 1:1 mixture of H_2_O and acetonitrile in pure acetonitrile or in pure methanol. The MSD1100 mass detector was utilized under the following conditions: mass range 100-2500 uma, positive scanning, energy of fragmentor 50 V, drying gas flow (nitrogen) 10.0 mL/min, nebulizer pressure 45 psig, drying gas temperature 350 °C, capillary voltage 4500 V. Optical rotations were measured on a Perkin Elmer 341 polarimeter. Column chromatography was performed using Kieselgel 60 Merck (230-400 mesh ASTM). 

*(4S,5R)-1-(3,4-Dimethyl-2-oxo-5-phenylimidazolidine)carboxamide* (**3**): The imidazolidin-2-one **1** (0.38 g; 2.0 mmol) was dissolved in dry THF (3 mL) and n-BuLi (0.74 mL of 2.7 M solution in hexanes; 2.0 mmol) was added at 0 °C and after 1 h the solution was slowly added dropwise at -78 °C to phosgene (1.98 mL of 1.98 M solution in toluene; 4.0 mmol) to give compound **2**, which was not isolated. After 3 h 30% aqueous NH_4_OH (1.2 mL) was added to the mixture which after 30 min was extracted with DCM (2 × 40 mL). After drying (Na_2_SO_4_), the solvent was removed under reduced pressure, to give compound **3** as a white solid (0.42 g; 90% yield), which was used without further purification. ^1^H-NMR: δ 0.80 (d, *J* = 6.6 Hz, 3H, 4-CH_3_), 2.80 (s, 3H, 3-CH_3_), 3.91 (dq, *J* = 6.6 Hz, *J* = 9.3 Hz, 1H, H-4), 4.99 (br s, 1H, NH), 5.27 (d, *J* = 9.3 Hz, 1H, H-5), 7.13 - 7.17 (m, 2 ArH), 7.22 - 7.38 (m, 3 ArH) 8.11 (br s, 1H, NH); ^13^C-NMR: δ 14.8, 28.2, 54.5, 59.1, 126.8, 128.1, 128.5, 136.7, 153.1 157.4; [α]_D_ -3.7 (c 0.5, CHCl_3_) (Lit. [19]: -2.3 c 1, CH_2_Cl_2_); MS (ESI): *m/z* 233.1 [M]^+^, 256.1 [M+Na]^+^; Anal. calcd. for C_12_H_15_N_3_O_2_: C, 61.79; H, 6.48; N, 18.01. Found: C, 61.74; H, 6.54; N, 17.96.

### Preparation of derivatives ***6*** and ***7***

To a solution containing compound **3** (1.17 g; 5.0 mmol) in dichloroethane (10 mL), oxalyl chloride (0.66 g; 5.2 mmol) was added and the mixture was refluxed for 4 h. The solvent was partially removed and then the Baylis-Hillman adducts **5a-f** (3.3 mmol) were added, dissolved in DCM (10 mL). The reaction was stirred for 12 h, volatiles were removed under reduced pressure and the products **6** and **7** were obtained as equimolar mixtures hard to separate by silica gel chromatography (cyclohexane-ethyl acetate 1:1), although small amounts could be obtained pure enough for analytical determinations.

*Methyl(1'S,4"S,5"R)-2-[(3",4"-dimethyl-2"-oxo-5"-phenylimidazolidine-1"-carbonylcarbamoyloxy)-(phenyl)methyl]acrylate* (**6a**) *and its (1'R,4"S,5"R)-isomer* **7a**: The title compounds were obtained in 54% overall yield as an equimolar diastereomeric mixture. White solid; MS (ESI): *m/z* 451.2 [M^+^], 474.2 [M+Na]^+^; Anal. Calcd. for C_24_H_25_N_3_O_6_: C, 63.85; H, 5.58; N, 9.31; Found: C, 63.79; H, 5.54; N, 9.35. *Isomer* **6a:** *R_f_* 0.63 (cyclohexane-AcOEt 1:1); ^1^H-NMR: δ 0.82 (d, *J* = 6.6 Hz, 3H, 4"-CH_3_), 2.82 (s, 3H, 3"-CH_3_), 3.68 (s, 3H, OCH_3_), 3.95 (dq, *J* = 6.6 Hz, *J* = 8.7 Hz, 1H, H-4"), 5.29 (d, *J* = 8.7 Hz, 1H, H-5"), 5.97 (bs, 1H, H-1'), 6.41 (s, 1H, =CH_2_), 6.67 (s, 1H, =CH_2_), 7.07 - 7.18 (m, 2 ArH), 7.24 - 7.42 (m, 8 ArH), 10.98 (s, 1H, NH); ^13^C-NMR: δ 14.5, 28.0, 51.9, 54.3, 59.0, 74.4, 126.2, 126.8, 126.9, 127.7, 127.8, 128.3, 128.5, 128.7, 135.6, 137.3, 139.0, 147.2, 149.3, 156.8, 165.2. *Isomer* **7a:** *R_f_* 0.58 (cyclohexane-AcOEt 1:1); ^1^H-NMR: δ 0.82 (d, *J* = 6.6, 3H, 4"-CH_3_), 2.84 (s, 3H, 3"-CH_3_), 3.68 (s, 3H, OCH_3_), 3.96 (dq, *J* = 6.6 Hz, *J* = 8.8 Hz, 1H, H-4"), 5.31 (d, *J* = 8.8 Hz, 1H, H-5"), 5.97 (bs s, 1H, H-1'), 6.41 (s, 1H, =CH_2_), 6.68 (s, 1H, =CH_2_), 7.07 - 7.18 (m, 2 ArH), 7.23 - 7.44 (m, 8 ArH), 10.97 (s, 1H, NH); ^13^C-NMR: δ 14.6, 28.0, 51.9, 54.3, 59.1, 74.4, 126.0, 126.8, 126.9, 127.8, 128.4, 128.5, 128.6, 135.6, 139.0, 147.3, 149.3, 156.9, 165.2. 

*Ethyl(1'S,4"S,5"R)-2-[(3",4"-dimethyl-2"-oxo-5"-phenylimidazolidine-1"-carbonylcarbamoy-loxy)(4-chlorophenyl)methyl]acrylate* (**6b**) *and its (1'R,4"S,5"R)-isomer* **7b:** The title compounds were obtained in 50% overall yield as an equimolar diastereomeric mixture. White solid; MS (ESI): *m/z* 499.2 [M]^+^, 522.2 [M+Na]^+^; Anal. Calcd. for C_25_H_26_ClN_3_O_6_: C, 60.06; H, 5.24; N, 8.40; Found: C, 60.02; H, 5.19; N, 8.35. Isomer **6b:** *R_f_* 0.67 (cyclohexane-AcOEt 1:1); ^1^H-NMR: δ 0.82 (d, *J* = 6.7 Hz, 3H, 3"-CH_3_), 1.19 (t, *J* = 7.2 Hz, 3H, CH_3_CH_2_O), 2.83 (s, 3H, 3"-CH_3_), 3.96 (dq, *J* = 6.7 Hz, *J* = 8.8 Hz, 1H, H-4"), 4.13 (q, *J* = 7.2 Hz, 2H, CH_3_CH_2_O), 5.29 (d, *J* = 8.8 Hz, 1H, H-5"), 5.98 (s, 1H, H-1'), 6.42 (s, 1H, =CH_2_), 6.62 (s, 1H, =CH_2_), 7.07 - 7.17 (m, 2 ArH), 7.21 - 7.86 (m, 8 ArH), 11.00 (s, 1H, NH); ^13^C-NMR: δ 13.9, 14.6, 28.0, 54.4, 59.1, 61.0, 73.9, 126.0, 126.9, 128.4, 128.5, 128.6, 129.3, 134.3, 135.6, 136.1, 136.9, 147.2, 149.2, 156.9, 164.6. *Isomer* **7b:** *R_f_* 0.61 (cyclohexane-AcOEt 1:1); ^1^H-NMR: δ 0.83 (d, *J* = 6.6 Hz, 3H, 3"-CH_3_), 1.23 (t, *J* = 7.1 Hz, 3H, CH_3_CH_2_O), 2.84 (s, 3H, 3"-CH_3_), 3.97 (dq, *J* = 6.6 Hz, *J* = 8.7 Hz, 1H, H-4"), 4.13 (q, *J* = 7.1 Hz, 2H, CH_3_CH_2_O), 5.30 (d, *J* = 8.7 Hz, 1H, H-5"), 5.97 (s, 1H, H-1'), 6.41 (s, 1H, =CH_2_), 6.63 (s, 1H, =CH_2_), 7.05 - 7.17 (m, 2 ArH), 7.21 - 7.38 (m, 8 ArH), 10.99 (s, 1H, NH); ^13^C-NMR: δ 14.0, 14.6, 28.1, 54.4, 59.2, 61.0, 73.9, 125.9, 126.9, 128.4, 128.6, 129.4, 134.4, 135.6, 136.0, 139.0, 147.3, 149.3, 156.9, 164.6. 

*Methyl (1'S,4"S,5"R)-2-[(3",4"-dimethyl-2"-oxo-5"-phenylimidazolidine-1"-carbonylcarbamoyloxy)-(4-nitrophenylmethyl)]acrylate* (**6c**) *and its (1'R,4"S,5"R)-isomer* (**7c**): The title compounds were obtained in 50% overall yield as an equimolar diastereomeric mixture. White solid; MS (ESI): *m/z* 496.2 [M]^+^, 519.2 [M+Na]^+^; Anal. Calcd. for C_24_H_24_N_4_O_8_: C, 58.06; H, 4.87; N, 11.28; Found: C, 58.00; H, 4.84; N, 11.33. *Isomer* **6c:** *R_f_* 0.56 (cyclohexane-AcOEt 1:1); ^1^H-NMR: δ 0.83 (d, *J* = 6.6 Hz, 3H, 4"-CH_3_), 2.84 (s, 3H, 3"-CH_3_), 3.69 (s, 3H, OCH_3_), 3.97 (dq, *J* = 6.6 Hz, *J* = 8.8 Hz, 1H, H-4"), 5.30 (d, *J* = 8.8 Hz, 1H, H-5"), 6.08 (d, *J* = 1.3 Hz, 1H, H-1'), 6.47 (s, 1H, =CH_2_), 6.70 (s, 1H, =CH_2_), 7.08 - 7.18 (m, 2 ArH), 7.25 - 7.35 (m, 3 ArH), 7.58 (d, J = 8.9 Hz, 2 ArH), 8.16 (d, *J* = 8.9 Hz, 2 ArH), 11.09 (s, 1H, NH); ^13^C-NMR: δ 14.6, 26.9, 28.1, 52.1, 54.4, 59.2, 73.5, 123.7, 126.9, 127.3, 128.5, 128.7, 135.6, 138.1, 144.7, 147.2, 147.9, 149.2, 156.9, 164.8. *Isomer* **7c:** *R_f_* 0.49 (cyclohexane-AcOEt 1:1); ^1^H-NMR: δ 0.83 (d, *J* = 6.6 Hz, 3H, 4"-CH_3_), 2.84 (s, 3H, 3"-CH_3_), 3.69 (s, 3H, OCH_3_), 3.98 (dq, *J* = 6.6 Hz, *J* = 8.8 Hz, 1H, H-4"), 5.30 (d, *J* = 8.8 Hz, 1H, H-5"), 6.06 (d, *J* = 1.1 Hz, 1H, H-1'), 6.46 (s, 1H, =CH_2_), 6.71 (s, 1H, =CH_2_), 7.08 - 7.18 (m, 2 ArH), 7.21 - 7.35 (m, 3 ArH), 7.58 (d, *J* = 8.8 Hz, 2 ArH), 8.17 (d, *J* = 8.8 Hz, 2 ArH), 11.09 (s, 1H, NH); ^13^C-NMR: δ 14.6, 26.9, 28.1, 52.1, 54.4, 59.2, 73.5, 123.7, 126.8, 126.9, 127.2, 127.8, 128.1, 128.5, 128.7, 135.5, 138.1, 144.6, 147.2, 147.8, 149.2, 156.9, 164.8. 

*Ethyl (1'S,4"S,5"R)-2-[(3",4"-dimethyl-2"-oxo-5"-phenylimidazolidine-1"-carbonylcarbamoyloxy)-(naphthalen-1-ylmethyl)]acrylate* (**6d**) *and its (1'R,4"S,5"R)-isomer* (**7d**): The title compounds were obtained in 60% overall yield as an equimolar diastereomeric mixture. White solid; MS (ESI): *m/z* 515.2 [M]^+^, 538.2 [M+Na]^+^; Anal. Calcd. for C_29_H_29_N_3_O_6_: C, 67.56; H, 5.67; N, 8.15; Found: C, 67.51; H, 5.62; N, 8.19. *Isomer* **6d:** *R_f_* 0.62 (cyclohexane-AcOEt 1:1); ^1^H-NMR: δ 0.80 (d, *J* = 6.6 Hz, 3H, 4"-CH_3_), 1.17 (t, *J* = 7.1 Hz, 3H, CH_3_CH_2_O), 2.81 (s, 3H, 3"-CH_3_), 3.92 (dq, *J* = 6.6 Hz, *J* = 8.8 Hz, 1H, H-4"), 4.12 (q, *J* = 7.1 Hz, 2H, CH_3_CH_2_O), 5.28 (d, *J* = 8.8 Hz, H-5"), 6.02 (d, *J* = 1.3 Hz, 1H, H-1'), 6.46 (s, 1H, =CH_2_), 6.85 (s, 1H, =CH_2_), 7.09 - 7.15 (m, 2 ArH), 7.21 - 7.38 (m, 3ArH), 7.40 - 7.51 (m, 3ArH), 7.74 - 7.87 (m, 4ArH), 11.02 (s, 1H, NH); ^13^C-NMR: δ 14.0, 14.6, 26.9, 28.0, 54.4, 59.1, 60.9, 74.7, 125.3, 126.1, 126.3, 127.0, 127.4, 127.6, 128.1, 128.2, 128.4, 128.6, 133.0, 133.3, 134.8, 135.7, 139.3, 147.3, 149.4, 156.9, 164.8. *Isomer* **7d:** *R_f_* 0.56 (cyclohexane-AcOEt 1:1); ^1^H- NMR: δ 0.76 (d, *J* = 6.6 Hz, 3H, 4"-CH_3_), 1.18 (t, *J* = 7.1 Hz, 3H, CH_3_CH_2_O), 2.79 (s, 3H, 3"-CH_3_), 3.90 (dq, *J*= 6.6 Hz, *J* = 8.8 Hz, 1H, H-4"), 4.12 (q, *J* = 7.1 Hz, 2H, CH_3_CH_2_O), 5.28 (d, *J* = 8.8 Hz, 1H, H-5"), 6.05 (s, 1H, H-1'), 6.47 (s, 1H, =CH_2_), 6.88 (s, 1H, =CH_2_), 7.05 -7.16 (m, 2 ArH), 7.20 - 7.32 (m, 3 ArH), 7.40 - 7.55 (m, 3 ArH), 7.75 - 7.91 (m, 4 ArH), 11.07 (s, 1H, NH); ^13^C-NMR: δ 13.8, 14.4, 26.8, 27.9, 54.2, 59.0, 60.8, 74.6, 125.2, 125.8, 126.0, 126.2, 126.8, 127.3, 127.5, 128.1, 128.2, 128.4, 132.9, 133.2, 134.6, 135.6, 139.2, 147.2, 149.3, 156.7, 164.6. 

*Methyl (1'R,4"S,5"R)-2-[(3",4"-dimethyl-2"-oxo-5"-phenylimidazolidine-1"-carbonylcarbamoyloxy)-(ethoxycarbonylmethyl]acrylate* (**6e**) *and its (1'S,4"S,5"R)-isomer* (**7e**): The title compounds were obtained in 77% overall yield as an equimolar diastereomeric mixture. White solid; MS (ESI): *m/z* 447.2 [M]^+^, 470.2 [M+Na]^+^; Anal. Calcd. for C_21_H_25_N_3_O_8_: C, 56.37; H, 5.63; N, 9.39; Found: C, 56.33; H, 5.57; N, 9.43. *Isomer* **6e**: *R_f_* 0.57 (cyclohexane-AcOEt 1:1); ^1^H-NMR: δ 0.80 (d, *J* = 6.6, 3H, 4"-CH_3_), 1.21 (t, *J* = 7.0 Hz, 3H, CH_3_CH_2_O), 2.80 (s, 3H, 3"-CH_3_), 3.77 (s, 3H, OCH_3_), 3.93 (dq, *J* = 6.6, *J* = 8.7, 1 H, H-4"), 4.17 (q, *J* = 7.0 Hz, 2H, CH_3_CH_2_O), 5.29 (d, *J* = 8.7, 1H, H-5"), 5.94 (s, 1H, H-1'), 6.00 (s, 1H, =CH_2_), 6.47 (s, 1H, =CH_2_), 7.06 – 7.21 (m, 3 ArH), 7.22 – 7.93 (m, 7 ArH + NH); + NH); ^13^C-NMR:: δ 14.5, 28.0, 51.9, 54.3, 59.0, 74.4, 126.2, 126.9, 127.7, 127.8, 128.3, 128.5, 128.7, 135.6, 137.3, 139.0, 147.2, 149.3, 156.8, 165.2. *Isomer* **7e**: *R_f_* 0.51 (cyclohexane-AcOEt 1:1); ^1^H-NMR: δ 0.83 (d, *J* = 6.6, 3H, 4"-CH_3_), 1.22 (t, *J* = 7.0 Hz, 3H, CH_3_CH_2_O), 2.81 (s, 3H, 3"-CH_3_), 3.76 (s, 3H, OCH_3_), 3.95 (dq, *J* = 6.6, *J* = 8.8, 1H, H-4"), 4.19 (q, *J* = 7.0 Hz, 2H, CH_3_CH_2_O), 5.31 (d, *J* = 8.8, 1H, H-5"), 5.95 (s, 1H, H-1'), 5.99 (s, 1H, =CH_2_), 6.48 (s, 1H, =CH_2_), 7.07 – 7.20 (m, 3 ArH), 7.26 – 7.47 (m, 7 ArH + NH); ^13^C-NMR: δ 14.6, 28.0, 51.9, 54.3, 59.1, 74.4, 126.0, 126.8, 126.9, 127.8, 128.4, 128.5, 128.6, 135.6, 139.0, 147.3, 149.3, 156.8, 165.2. 

*Methyl (3S,4'S,5'R)-3-(3',4'-dimethyl-2'-oxo-5'-phenylimidazolidine-1'-carbonylcarbamoyloxy)-5-methyl-2-methylenehexanoate* (**6f**) *and its (3R,4'S,5'R)-isomer* (**7f**): The title compounds were obtained in 56% overall yield as an equimolar diastereomeric mixture. White solid; MS (ESI): *m/z* 431.2 [M]^+^, 454.2 [M+Na]^+^; Anal. Calcd. for C_22_H_29_N_3_O_6_: C, 61.24; H, 6.77; N, 9.74. Found: C, 61.18; H, 6.73; N, 9.69. *Isomer* **6f**: *R_f_* 0.53 (cyclohexane-AcOEt 1:1); ^1^H-NMR: δ 0.81 (d, *J* = 6.6, 3H, CH_3_), 0.90 (d, *J* = 6.6, 3H, CH_3_), 0.93 (d, *J* = 6.6, 3H, 4'-CH_3_), 1.45 – 1.73 (m, 3H, CH-CH_2_), 2.83 (s, 3H, 3'-CH_3_), 3.73 (s, 3H, OCH_3_), 3.96 (dq, *J* = 6.6, *J* = 8.8, 1H, H-4'), 5.30 (d, *J* = 8.8, 1H, H-5'), 5.66 (dd, *J* = 4.0, *J* = 8.8, 1H, H-3), 5.82 (s, 1H, =CH_2_), 6.25 (s, 1H, =CH_2_), 7.09 – 7.18 (m, 3 ArH), 7.26 – 7.35 (m, 2 ArH + NH); ^13^C NMR (50 MHz, CDCl_3_) δ 14.5, 21.6, 23.1, 24.7, 26.8, 29.1, 43.7, 51.8, 54.3, 59.1, 72.0, 125.3, 126.9, 128.3, 128.5, 135.7, 140.3, 147.3, 149.7, 156.9, 165.5. Isomer **7f**: *R_f_* 0.44 (cyclohexane-AcOEt 1:1);^ 1^H-NMR: δ 0.79 (d, *J* = 6.6, 3H, CH_3_), 0.88 (d, *J* = 5.3, 3H, CH_3_), 0.91 (d, *J* = 5.3, 3H, 4'-CH_3_), 1.41 - 1.71 (m, 3H, CH-CH_2_), 2.81 (s, 3H, 3'-CH_3_), 3.73 (s, 3H, OCH_3_), 3.95 (dq, *J* = 5.3, *J* = 8.8, 1H, H-4'), 5.28 (d, *J* = 8.8, 1H, ), 5.65 (dd, *J* = 3.3, *J* = 8.9, 1H, H-3), 5.79 (s, 1H, =CH_2_), 6.24 (s, 1H, =CH_2_), 7.07 – 7.15 (m, 3 ArH), 7.23 – 7.35 (m, 2 ArH + NH); ^13^C-NMR: δ 14.6, 21.5, 23.1, 24.6, 26.8, 27.9, 43.7, 51.7, 54.3, 59.0, 71.8, 125.0, 126.9, 128.2, 128.5, 135.7, 140.4, 147.3, 149.6, 158.9, 165.5.

### Preparation of compounds ***8*** and ***9***

To a solution containing an equimolar mixture of acyl carbamates **6** and **7** (3.0 mmol) in dry DCM (8.0 mL), DABCO (0.07 g; 0.6 mmol) was slowly added at r.t. After 20 min, 2 M HCl (10 mL) was added and the mixture was extracted with DCM (2 × 35 mL). After drying (Na_2_SO_4_) and removal of the volatiles, the residue was purified by silica gel chromatography (cyclohexane:ethyl acetate 60:40), to give pure compounds **8** and **9**.

*Methyl (1'S,4"S,5"R)-2-[(3",4"-dimethyl-2"-oxo-5"-phenylimidazolidine-1"-carboxamido)(phenyl)-methyl]acrylate* (**8a**) *and its (1'R,4"S,5"R)-isomer* (**9a**): The title compounds were obtained in 60% overall yield as 75:25 diastereomeric mixture. MS (ESI): *m/z* 407.2 [M]^+^, 430.2 [M+Na]^+^; Anal. Calcd. for C_23_H_25_N_3_O_4_: C, 67.80; H, 6.18; N, 10.31; Found: C, 67.84; H, 6.13; N, 10.27. *Isomer* **8a**: *R_f_* 0.47 (cyclohexane-AcOEt 1:1); White crystals; Mp 50-52 °C; ^1^H-NMR: δ 0.80 (d, *J* = 6.6 Hz, 3H, 4"-CH_3_), 2.80 (s, 3H, 3"-CH_3_), 3.64 (s, 3H, OCH_3_), 3.91 (dq, *J* = 6.6 Hz, *J*= 8.8 Hz, 1H, H-4"), 5.26 (d, *J* = 8.7 Hz, 1H, H-1'), 5.82 (s, 1H, =CH_2_), 5.89 (d, *J* = 8.8 Hz, 1H, H-5"), 6.29 (s, 1H, =CH_2_), 7.11 - 7.19 (m, 2 ArH), 7.20 - 7.41 (m, 8 ArH), 9.21 (d, *J* = 8.7, 1H, NH); ^13^C-NMR: δ 14.7, 26.9, 28.0, 51.8, 54.5, 54.8, 59.3, 125.7, 126.8, 126.9, 127.4, 128.0, 128.5, 136.8, 140.1, 140.3, 151.6, 157.9, 165.9; [α]_D_ -122.0 (c 0.5, CHCl_3_). *Isomer* **9a**: *R_f_* 0.27 (cyclohexane-AcOEt 1:1); White crystals; Mp 123-125 °C; ^1^H-NMR: δ 0.81 (d, *J* = 6.6 Hz, 3H, 4"-CH_3_), 2.81 (s, 3H, 3"-CH_3_), 3.66 (s, 3H, OCH_3_), 3.94 (dq, *J* = 6.6 Hz, *J* = 8.8 Hz, 1H, H-4"), 5.28 (d, *J* = 8.5 Hz, 1H, H-1'), 5.89 (d, *J* = 8.8 Hz, 1H, H-5"), 5.91 (s, 1H, =CH_2_), 6.36 (s, 1H, =CH_2_), 7.08 - 7.17 (m, 2 ArH), 7.20 - 7.39 (m, 8 ArH), 9.15 (d, *J* = 8.5 Hz, 1H, NH); ^13^C- NMR: δ 14.8, 26.9, 28.0, 51.8, 54.6, 54.9, 59.4, 126.1, 126.7, 126.8, 127.0, 127.3, 127.8, 128.1, 128.3, 128.5, 128.6, 136.8, 139.6, 140.6, 151.8, 157.9, 165.9; [α]_D_ 93.0 (c 0.5, CHCl_3_). 

*Ethyl (1'S,4"S,5"R)-2-[(3",4"-dimethyl-2"-oxo-5"-phenylimidazolidine-1"-carboxamido)(4-chloro-phenyl)methyl]acrylate* (**8b**) *and its (1'R,4"S,5"R)-isomer* (**9b**): The title compounds were obtained in 63% overall yield as 76:24 diastereomeric mixture. MS (ESI): *m/z* 455.2 [M]^ +^, 478.2 [M+Na]^+^; Anal. Calcd. for C_24_H_26_ClN_3_O_4_: C, 63.22; H, 5.75; N, 9.22; Found: C, 63.17; H, 5.71; N, 9.26. *Isomer* **8b**: *R_f_* 0.47 (cyclohexane-AcOEt 1:1); White crystals; Mp 46-48 °C; ^1^H-NMR: δ 0.80 (d, *J* = 6.6 Hz, 3H, 4"-CH_3_), 1.16 (t, *J* = 7.2 Hz, 3H, CH_3_CH_2_O), 2.80 (s, 3H, 3"-CH_3_), 3.94 (dq, *J* = 6.6 Hz, *J*= 8.8 Hz, 1H, H-4"), 4.09 (50% q, *J* = 7.2 Hz, 2H, CH_3_CH_2_O), 4.10 (50% q, *J* = 7.2 Hz, 2H, CH_3_CH_2_O), 5.26 (d, *J* = 8.3 Hz, 1H, H-1'), 5.81 (s, 1H, =CH_2_), 5.86 (d, *J* = 8.8 Hz, 1H, H-5"), 6.31 (s, 1H, =CH_2_), 7.10 - 7.18 (m, 2 ArH), 7.22 - 7.38 (m, 7 ArH), 9.22 (d, *J* = 8.3 Hz, 1H, NH); ^13^C-NMR: δ 13.9, 14.8, 26.9, 28.1, 54.4 (50%), 54.6 (50%), 59.3, 61.0, 125.9, 126.8, 128.1, 128.3, 128.4, 128.5, 128.6, 133.2, 136.8, 139.0, 140.2, 151.7, 157.9, 165.3; [α]_D_ -125.0 (c 0.5, CHCl_3_). *Isomer* **9b**: *R_f_* 0.29 (cyclohexane-AcOEt 1:1); Low melting white solid; ^1^H-NMR: δ 0.80 (d, *J* = 6.6 Hz, 3H, 4"-CH_3_), 1.17 (t, *J* = 7.2 Hz, 3H, CH_3_CH_2_O), 2.80 (s, 3H, 3"-CH_3_), 3.93 (dq, *J* = 6.6 Hz, *J* = 8.8 Hz, 1H, H-4"), 4.10 (q, *J* = 7.2 Hz, 3H, CH_3_CH_2_O), 5.26 (d, *J* = 8.8 Hz, 1H, H-1'), 5.86 (d, 1H, *J* = 8.8 Hz, 1H, H-5"), 5.89 (s, 1H, =CH_2_), 6.37 (s, 1H, =CH_2_), 7.09 - 7.18 (m, 2 ArH), 7.19 - 7.38 (m, 7 ArH), 9.17 (d, *J* = 8.8, 1H, NH); ^13^C-NMR: δ 13.9, 14.8, 28.0, 29.7, 54.4 (50%), 54.6 (50%), 59.4, 60.9, 126.4, 126.8, 128.1, 128.2, 128.5, 133.1, 136.8, 138.5, 140.3, 151.8, 157.9, 165.3; [α]_D_ 155.0 (c 0.5, CHCl_3_). 

*Methyl (1'S,4"S,5"R)-2-[(3",4"-dimethyl-2"-oxo-5"-phenylimidazolidine-1"-carboxamido)(4-nitro-phenyl)methyl]acrylate* (**8c**) *and its (1'R,4"S,5"R)-isomer* (**9c**): The title compounds were obtained in 61% overall yield as 70:30 diastereomeric mixture. MS (ESI): *m/z* 452.2 [M]^+^, 475.2 [M+Na]^+^; Anal. Calcd. for C_23_H_24_N_4_O_6_: C, 61.05; H, 5.35; N, 12.38; Found: C, 61.00; H, 5.31; N, 12.35. *Isomer* **8c**: *R_f_* 0.50 (cyclohexane-AcOEt 1:1); White crystals; Mp. 55-57 °C; ^1^H-NMR: δ 0.82 (d, *J* = 6.6 Hz, 3H, 4"-CH_3_), 2.83 (s, 3H, 3"-CH_3_), 3.68 (s, 3H, OCH_3_), 3.95 (dq, *J* = 6.6 Hz, *J* = 8.8 Hz, 1H, H-4"), 5.26 (d, *J* = 8.7 Hz, 1H, H-1'), 5.92 (s, 1H, =CH_2_), 5.95 (d, *J* = 8.8 Hz, 1H, H-5"), 6.38 (s, 1H, =CH_2_), 7.10 - 7.22 (m, 2 ArH), 7.25 - 7.41 (m, 3 ArH), 7.51 (d, *J*= 8.5 Hz, 2 ArH), 8.16 (d, *J* = 8.5 Hz, 2 ArH), 9.44 (d, *J* = 8.7 Hz, 1H, NH); ^13^C-NMR: δ 14.8, 28.1, 52.1 (50%), 52.2 (50%), 54.6 (50%), 54.8 (50%), 59.4, 76.4, 123.8, 126.8, 127.5, 127.8, 128.2, 128.6, 136.7, 139.1, 147.8, 151.9, 157.9, 165.5; [α]_D_ -107.0 (c 0.5, CHCl_3_). *Isomer* **9c**: *R_f_* 0.20 (cyclohexane-AcOEt 1:1); White crystals; Mp. 62-64 °C; ^1^H-NMR: δ 0.83 (d, *J* = 6.6 Hz, 3H, 4"-CH_3_), 2.83 (s, 3H, 3"-CH_3_), 3.70 (s, 3H, OCH_3_), 3.96 (dq, *J* = 6.6 Hz, *J* = 8.8 Hz, 1H, H-4"), 5.28 (d, *J* = 8.8 Hz, 1H, H-1'), 5.97 (s, 1H, =CH_2_), 5.97 (d, *J* = 8.5 Hz, 1H, H-5"), 6.43 (s, 1H, =CH_2_), 7.11 - 7.18 (m, 2 ArH), 7.25 - 7.38 (m, 3 ArH), 7.44 (d, *J* = 8.5 Hz, 2 ArH), 8.13 (d, *J* = 8.5 Hz, 2 ArH), 9.37 (d, *J* = 8.8 Hz, 1H, NH); ^13^C-NMR: δ 14.8, 28.1, 52.1, 54.6 (50%), 54.8 (50%), 59.5, 76.4, 123.7, 126.8, 127.4, 128.0, 128.3, 128.6, 136.7, 139.3, 147.4, 152.0, 156.2 (50%), 157.8 (50%), 165.4; [α]_D_ 78.9 (c 0.5, CHCl_3_). 

*Ethyl (1'S,4"S,5"R)-2-[(3",4"-dimethyl-2"-oxo-5"-phenylimidazolidine-1"-carboxamido) (naphthalen-1-yl)methyl]acrylate* (**8d**) and its (1'*R*,4"*S*,5"*R*)-isomer (**9d**): The title compounds were obtained in 58% overall yield as 70:30 diastereomeric mixture. MS (ESI): *m/z* 471.2 [M]^+^, 494.2 [M+Na]^+^; Anal. Calcd. for C_28_H_29_N_3_O_4_: C, 71.32; H, 6.20; N, 8.91. Found: C, 71.36; H, 6.15; N, 8.87. *Isomer* **8d**: *R_f_* 0.50 (cyclohexane-AcOEt 1:1); White crystals; Mp. 53-55 °C; ^1^H-NMR: δ 0.79 (d, *J* = 6.6 Hz, 3H, 4"-CH_3_), 1.13 (t, *J* = 7.1 Hz, 3H, CH_3_CH_2_O), 2.79 (s, 3H, 3"-CH_3_), 3.88 (dq, *J* = 6.6 Hz, *J* = 8.8 Hz, 1H, H-4"), 4.08 (q, *J* = 7.1 Hz, 2H, CH_3_CH_2_O), 5.28 (d, *J* = 8.5 Hz, 1H, H-1'), 5.90 (s, 1H, =CH_2_), 6.10 (d, *J* = 8.8 Hz, 1H, H-5"), 6.38 (s, 1H, =CH_2_), 7.15 - 7.21 (m, 2 ArH), 7.25 - 7.52 (m, 6 ArH), 7.74 - 7.86 (m, 4 ArH), 9.31 (d, *J* = 8.5, 1H, NH); ^13^C-NMR: δ 13.9, 14.7, 28.0, 54.5, 55.0, 59.3, 60.8, 125.3, 125.6, 125.7, 125.8, 126.0, 126.8, 127.4, 127.6, 127.7, 128.0, 128.3, 128.5, 132.8, 133.4, 137.0, 137.8, 140.6, 151.7, 157.9, 165.5; [α]_D_ -106.0 (c 0.5, CHCl_3_). *Isomer* **9d**: *R_f_* 0.35 (cyclohexane-AcOEt 1:1); White crystals; Mp. 56-57 °C; ^1^H-NMR: δ 0.79 (d, *J* = 6.6 Hz, 3H, 4"-CH_3_), 1.16 (t, *J* = 7.1 Hz, 3H, CH_3_CH_2_O), 2.80 (s, 3H, 3"-CH_3_), 3.91 (dq, *J* = 6.6 Hz, *J* = 8.8 Hz, 1H, H-4"), 5.32 (d, *J* = 8.8 Hz, 1H, H-1'), 6.00 (s, 1H, =CH_2_), 6.11 (d, *J* = 8.7 Hz, 1H, H-5"), 6.46 (s, 1H, =CH_2_), 7.14 - 7.20 (m, 2 ArH), 7.24 - 7.33 (m, 3 ArH), 7.39 - 7.49 (m, 3 ArH), 7.73 - 7.84 (m, 4 ArH), 9.29 (d, *J* = 8.7 Hz, 1H, NH); ^13^C-NMR: δ 13.8, 4.6, 27.9, 54.4, 54.8, 59.3, 60.7, 124.9, 125.4, 125.7, 125.9, 126.0, 126.7, 127.4, 127.9, 128.1, 128.4, 128.6, 132.6, 133.2, 136.8, 137.2, 140.7, 151.8 157.8, 165.4; [α]_D_ 54.0 (c 0.5, CHCl_3_). 

*Methyl (1'R,4"S,5"R)-2-[(3",4"-dimethyl-2"-oxo-5"-phenylimidazolidine-1"-carboxamido) (ethoxy-carbonyl)methyl]acrylate* (8e) *and its (1'S,4"S,5"R)-isomer* (**9e**): The title compounds were obtained in 77% overall yield as 75:25 diastereomeric mixture. MS (ESI): *m/z* 403.2 [M]^+^, 426.2 [M+Na]^+^; Anal. Calcd. for C_20_H_25_N_3_O_6_: C, 59.54; H, 6.25; N, 10.42; Found: C, 59.50; H, 6.21; N, 10.46. *Isomer* **8e**: *R_f_* 0.35 (cyclohexane-AcOEt 1:1); White crystals; Mp. 78-80 °C; ^1^H-NMR: δ 0.78 (d, *J* = 6.6 Hz, 3H, 4"-CH_3_), 1.21 (t, *J* = 7.1 Hz, 3H, CH_3_CH_2_O), 2.80 (s, 3H, 3"-CH_3_), 3.78 (s, 3H, OCH_3_), 3.88 (dq, *J* = 6.6 Hz, *J* = 8.4 Hz, 1H, H-4"), 4.15 (q, *J* = 7.1 Hz, 2H, CH_3_CH_2_O), 5.26 (d, *J* = 8.7 Hz, 1H, H-1'), 5.33 (d, *J* = 8.4 Hz, 1H, H-5"), 5.85 (s, 1H, =CH_2_), 6.31 (s, 1H, =CH_2_), 7.09 - 7.18 (m, 2 ArH), 7.22 - 7.35 (m, 3 ArH), 9.22 (d, *J* = 8.7 Hz, 1H, NH); ^13^C-NMR: δ 14.0, 14.7, 28.1, 52.1, 54.5, 54.6, 59.2, 61.7, 126.8, 128.0, 128.4, 129.3, 136.5, 136.8, 152.2, 157.6, 165.5, 169.7; [α]_D_ 17.8 (c 0.5, CHCl_3_). Isomer **9e**: *R_f_* 0.25 (cyclohexane-AcOEt 1:1); Low melting white solid; ^1^H-NMR: δ 0.80 (d, *J* = 6.6 Hz, 3H, 4"-CH_3_), 1.21 (t, *J* = 7.1 Hz, 3H, CH_3_CH_2_O), 2.81 (s, 3H, 3"-CH_3_), 3.79 (s, 3H, OCH_3_), 3.91 (dq, *J* = 6.6 Hz, *J*= 8.8 Hz, 1H, H-4"), 4.18 (q, *J* = 7.1 Hz, 2H, CH_3_CH_2_O), 5.23 (d, *J* = 8.8 Hz, 1H, H-5"), 5.28 (d, *J* = 8.2 Hz, 1H, H-1'), 5.92 (s, 1H, =CH_2_), 6.37 (s, 1H, =CH_2_), 7.10 - 7.21 (m, 2 ArH), 7.25 - 7.38 (m, 3 ArH), 9.25 (d, *J* = 8.2 Hz, 1H, NH); ^13^C-NMR: δ 14.0, 14.8, 28.1, 52.1, 54.5, 54.8, 59.4, 61.8, 126.9, 128.1, 128.5, 129.6, 136.8, 152.1, 157.6, 165.5, 169.5; [α]_D_ - 41.7 (c 0.5, CHCl_3_).
